# Awareness of occupational health hazards and occupational stress among dental care professionals: Evidence from the GCC region

**DOI:** 10.3389/fpubh.2022.922748

**Published:** 2022-09-08

**Authors:** Esra AlDhaen

**Affiliations:** Department of Management, Marketing and Information Systems, College of Business & Finance, Ahlia University, Manama, Bahrain

**Keywords:** occupational hazards, dental professionals, ergonomics, safe environment, GCC region

## Abstract

A hazardous work environment creates critical concerns, and resultantly, workers may suffer from job-related stress. So, this study aimed at identifying the nature of hazards prevailing in dental hospitals and their role in increasing job-related stress. The study also assumes that awareness of the existence of health hazards and their possible risk will originate the stress. To conduct the study, close-ended questionnaires were administered to 300 workers having more than 1 year of experience in Oral and Dental Health Services provided by the Kingdom of Bahrain. In total, 222 responses with an acceptable level of accuracy were included for statistical treatment. Results confirmed the prevalence of ergonomic, biological, physical, and, to some extent, chemical hazards in the workplace. Results revealed that stress befalls the employees as they know their exposure to these hazards. Ergonomic hazards have the highest prevalence, chemical hazards are the least prevalent, while biological and physical hazards fall in between. This study enriches the related bank of literature by tapping the hazards specifically in the dental hospitals' environment with the degree of intensity of their prevalence within the context at hand. The study of the impact of these workplace health hazards on occupational stress with mediating effect of awareness is also an addition to the existing literature. The findings may help hospital administrators to take correct measures to manage job-related stress that is counterproductive and take remedial steps to mitigate these hazards.

## Introduction

Prevalence of workplace safety issues is a common phenomenon in the world, and it is more serious in developing regions in particular. Many employees may get affected physically and mentally due to working in an unsafe work environment and may carry the consequences to their families and immediate social circles. An occupational hazard is an injury or ailment resulting from the work one does or from the surrounding in which one works (1–3). The consequences of workplace hazards could be trauma, even posttraumatic stress disorder (PTSD), loss of dignity, anxiety, depression, suicide attempt, decreased self-esteem, lack of trust in people, premature aging, losing autonomy, injuries, absenteeism, and physical and musculoskeletal injuries (4, 5).

The environment of a dental hospital is a complex setting. Medicines, chemicals, blood, waste disposal, laboratory, laundry, engineering, sanitation, maintenance, and other services enable the provision of dental care services (6). According to the literature on occupational hazards, research carried out on employees exposed to gold or mercury, mostly in dental hospitals, reveals that workplace risk from metallic and organic mercury exists in the ecosphere, and genetic elements are precarious in shaping resistance or risk sensitivity (4, 7). Sodium hypochlorite is usually used in endodontic therapy to dissolve organic elements and eradicate microbes (6, 7). Musculoskeletal complications are common among the employees of dental hospitals (8, 9). The effect of workplace hazards begins with the entry of a student into a dental college, with 79% of students complaining of back or neck pain at the undergraduate level in UK dental schools (8, 10). However, the (11) reported that the effect of ergonomic involvement in managing musculoskeletal illnesses among dental professionals is insignificant (11). The predominant sources of biological hazards are injury due to needle prick (80%) followed by the risk of contaminated substances (75%), whereas the most prevalent non-biological risks are back-ached (79%) followed by extra work hours (72%) (12, 13).

Working in a dental hospital is a stressful occupation. Curing and caring for distressful patients, increasing workload, and a hazardous work environment consistently make service providers stressed (14, 15). Stress itself is an emotional, mental, or physical factor that produces mental or physical strain. Occupational stress is psychosomatic stress related to one's job (16). Workplace stress usually comes from demands that don't match a person's abilities, knowledge, and skills (10). What one perceives as a threat or a danger can be perceived as a challenge or motivation by someone else (17). Work-related stress is common in dental hospitals and may compromise both the health of the staff working at health services and the quality of the work for the patients they serve (5). The corresponding productivity losses have economic implications for the employer of a health service. When occupational stress is caused, for example, by a physical agent, it is paramount to eradicate it at the source(s) (18). Studies have been carried out to show that employee awareness of occupational hazards positively affects employee stress levels; however, it is seen that stress levels are more in employees who have experienced hazards at work (19). According to a study, employees are less likely to experience work-related stress when demands and pressures of work are matched to their knowledge and abilities, control can be exercised over their work and the way they do it, support is received from supervisors and colleagues, and participation in decisions that concern their jobs is provided (10, 17).

The aim of this study is the context (Bahrain) where, to the best of our knowledge, such kind of research is scarce. The context is not identical to others in terms of infrastructure, resources, human development, and culture. Being in an emerging country, dental hospitals in Bahrain are possibly more prone to environmental hazards, and the workforce is more vulnerable. The study aims at identifying the prevailing hazards and their degree of intensity in dental hospitals. The study also aims to test whether the prevalence of workplace hazards creates work-related stress and whether employees are aware of workplace hazards and their consequences are more stressful. The analysis of mediation in the model is a somewhat novel addition to the literature. So, this study aimed to assess whether occupational hazards at the workplace cause occupational stress and whether awareness regarding occupational hazards mediates occupational stress.

## Theory and hypotheses

The relations of stress with occupational hazards and employee awareness have been explained by various theories. The Stress Concept Theory states that the resistance or vulnerability of an employee who is exposed to a stressful stimulus that hosts resistance is a crucial factor in the outcome of stress or the effect of stress on health (20, 21). Two factors are central in defining the intensity of a person's host resistance: the capacity to cope and social support (20, 22). Accident Theory that unifies productivity and safety together defines risk as a phenomenon attached to negative outcomes such as loss, damage, and regret (23, 24). In workplace health and safety (WHS) management, it is produced by the incidence of hazards that may generate harmful consequences such as injury or damage to property/environment (25, 26). Likewise, the Domino Theory of Safety says that it is the series of happenings that leads to an incident (27). The possible injury occurs as a result of an injury (Final Domino). An accident only occurs as a consequence of a mechanical or personal hazard. Hazards only arise as a result of the faults of people. People's faults are inherited and educated (22, 28). So, elimination of a visual domino caused the effect not to happen, and it is possible by training employees and making them aware of hazards in the work environment (29, 30). The ABC Theory states that the attitude, behavior, and conditions that follow due to risk factors encountered result in a change of behavior. In fact, everyone is motivated differently, and thus, understanding safety motivation in individuals becomes critical for long-term change of behavior (21, 29). The theory states that the typical hazards are structural, biological, mechanical, electrical, chemical, and physical hazards (29, 31).

Since this article focuses on the effect of occupational hazards on occupational stress with mediating role of employee awareness, Accident Theory, Domino Theory of Safety, and Stress Concept Theory provide a basis for this study because they state that to minimize hazards in the workplace, they need to be identified and eliminated. Risks include mechanical, chemical, and psychosocial hazards. Domino Theory states that hazards at the workplace can be minimized by staff training and being aware of their surroundings. Stress Concept Theory states that host factors need to be taken into account when assessing stress. Our assumption focuses on making employees aware of policies and procedures at the workplace to reduce occupational hazards encountered.

### Conceptual definition of variables

#### Occupational hazard

Occupational hazard is the independent variable, which includes chemical, physical, biological, and psychosocial hazards. These hazards are the potential causes of injuries in the workplace.

*Chemical hazards* include questions on dental allergies and eye/mouth splashes or injuries (32).

*Biological hazards* include questions on needle stick/sharps injuries leading to infectious diseases like HIV/Hepatitis (33).

**Ergonomics** include musculoskeletal injuries. Questions were related to back pain and sprains. Dentists are most prone to these injuries due to the posture in which they sit in dental chairs (34).

**Physical** hazards include questions on electric and safety wiring and physical obstacles at the workplace (35).

#### Occupational stress

Occupational stress is the dependent variable. It is defined as a cognitive state that occurs when the demands of a job are not aligned with the capabilities, knowledge, resources, and needs of the employee (36).

#### Employee awareness

Employee awareness is the mediating variable that explains the relationship between occupational hazards and stress (37). Employee awareness refers to the degree of employee knowledge and behavior related to workplace health and safety (38).

### Hypotheses

Keeping in view the underlying assumption of Stress Concept Theory, it is stated that the vulnerability of employees who are exposed to undesirable environmental stimuli and the host resistance is a crucial factor in producing stress. In this scenario, dentists are exposed to workplace health hazards that stimulate stress in employees. Thus, this hypothesis is formulated:

*Hypothesis 1: Employees who are engaged in the treatment of patients in dental hospitals suffer from job-related stress due to the existence of workplace health hazards*.

ABC theory mainly helps us understand the meanings of our reactions to adversity. This promotes the belief that external conditions are cognitively evaluated, and consequently, specific mental and emotional reactions come into play. It is assumed that employees' awareness of the prevalence of workplace hazards will augment employees' job-related stress levels; however, the literature reveals that a large number of employees were not aware of the prevalence of health hazards in the workplace (36, 39, 40). It is also assumed that awareness of workplace health hazards will moderate the relationship between the prevalence of health hazards and the level of occupational stress. Thus, the following two hypotheses are developed:

*Hypothesis 2: The level of job-related stress increases as employees' awareness of the prevalence of workplace health hazards*.
*Hypothesis 3: Employees' awareness of workplace health hazards mediates the relationship between workplace hazards and occupational stress*.

**Figure 1:**
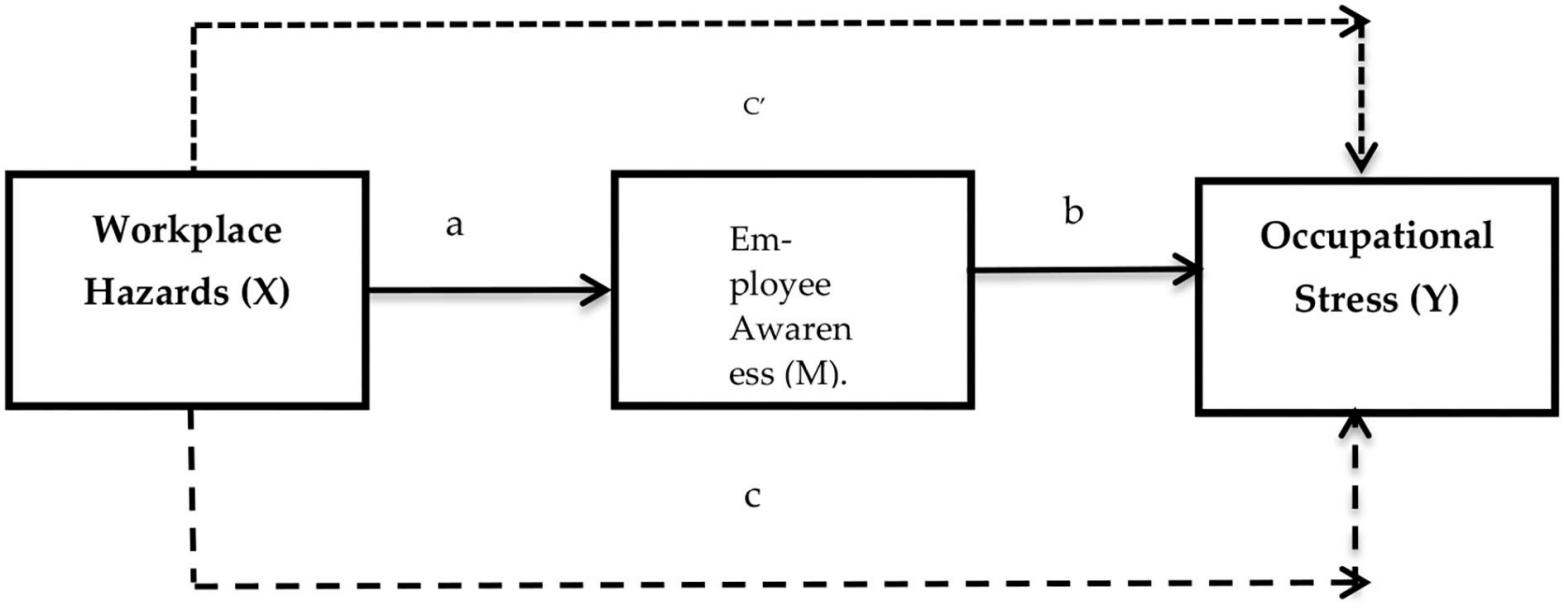
Proposed research model.

Figure 1 shows schematic view of the connection of variables and hypotheses.

## Methodology

### Research design

This is a quantitative, explanatory, and cross-sectional study. Survey design is used to gather data from the employees working in dental hospitals in the metropolitan city of Bahrain. Due to patient overpopulation, health hazards are likely to increase. Data were gathered from the employees associated with Oral and Dental Health Services, managed by the Ministry of Health, Kingdom of Bahrain. Three hundred healthcare workers having more than 1 year of experience were randomly selected for the sample; 239 questionnaires were received back, and 222 questionnaires accurate from all respects were included for analysis. A list of 1,728 employees was provided as the total human resource strength working in oral and dental facilities. Thus, this list was used as a sample frame to randomly select the sample.

Before data collection, the authors contacted the administrators seeking permission to collect data from their employees. A written guarantee was submitted to the relevant body to maintain ethical standards during data collection. Furthermore, the author obtained an informed consent form from each respondent for voluntary participation in the survey. In the face of the COVID-19 pandemic hospital, administrators were kind enough to instruct their HR departments to administer questionnaires to their selected employees on behalf of the author. In this way, the stay of the author in hospital for several hours and personal contact with each employee were avoided, and observance of SOPs against pandemics was maintained. In this regard, each hospital nominated four persons for data collection. The author provided necessary brief training to them on how to collect data through questionnaires. This study was conducted as per the ethical guidelines given in Helsinki Declaration (41).

### Measurement of instrument

The scale (questionnaire) had forty-four items (questions) that were responded to on a five-point Likert-type scale; 21 items for occupational hazards, 12 items for employee awareness, and 10 items for job stress were in the questionnaire. A questionnaire for occupational hazards was adopted from Viragi et al. (42), employee awareness was taken from NIOSH (43), and job stress was adopted from HSE (44). Table 1 in Annexure exhibits variables and the questions that measure them. Since standard instruments were adopted with already determined reliability coefficient, the instruments were presumed to be reliable. For the sake of this study, internal reliability analysis tests were run again to establish the reliability of instruments. The reliability test also confirmed the instrument was reliable for all the variables. Cronbach's alpha and composite reliability for chemical hazard were 0.686 and 0.724, respectively; for physical hazard were 0.630 and 0.699, respectively; for biological hazard were 0.721 and 0.802, respectively; for ergonomics were 0.724 and 0.794, respectively; for awareness were 0.865 and 0.895, respectively; and for stress were 0.756 and 0.752, respectively. Since all the scores are beyond 0.65, they are considered to be reliable.

### Analysis of data

Inferential statistics were applied to analyze the data. Partial least square (PLS) was used for structural equation modeling (SEM). This method allows researchers to analyze structural components (path model) and measurement components (factor model) in one model simultaneously (45). So, SEM draws an all-inclusive picture of the validity, reliability, and causality (46).

Although the instrument was adopted with already verified reliability and validity, to be on the safer side, further tests were applied to establish the quality of the data. Besides Cronbach's alpha and composite reliability tests, the AVE test was used to check convergent validity. Discriminant validity was established using MTHT and Fornell-Larcker methods. Autocorrelation, multicollinearity, and common method bias (CBM) were also checked through different tests. All these tests confirmed that the data were free from any discrepancy. A latent variables correlation test was run to check the association of variables, while R Square was used to determine the collective effect of the independent variables on the dependent variable. Path coefficient (Regression) was applied to test the hypotheses, and indirect effects were applied to test mediation.

## Results

### Demographics and quality control

#### Composition of respondents

The demographics of the sample were 44.1% males and 55.9% females. According to positions, 182 were general dentists, 6 were associate professors/principals, 7 were assistant professors, 20 were demonstrators, and 5 were dental technicians. As per age details, 9% of the participants were aged <23 years, 60% were aged between 24 and 35 years, 25% were aged between 31 and 35 years, and 5% were aged between 41 and 57 years. For job experience, 70% of respondents had 1–2 years of experience, 20% had 2–6 years of experience, and 10% had 8–34 years of experience. According to the nature of the hospital, 70% of participants work in the private sector and 30% work in the government sector.

#### Reliability

Cronbach's alpha and composite reliability scores were used to determine the reliability of the instrument (shown in Table 2 in the Annexure). All the Cronbach's alpha values were higher than 0.7 indicating high internal consistency except for physical hazards 0.63 and chemical hazards 0.69 although which is close to 0.7 and hence can be considered reliable. The values of composite reliability ensured instrument reliability as they were around or above the cutoff value which was 0.70. VIF values confirm that the data used are free of multicollinearity and common method bias (CMB). The occurrence of VIF >10 indicates the existence of multicollinearity (47), while VIF values >3.3 are proposed as an indication that a model may be contaminated by common method bias. Therefore, if all the VIFs resulting from a full collinearity test are equal to or <3.3, the model will be considered free of common method bias (48). All the VIFs extracted from our data have values <3.3 as shown in Table 3 in the Annexure.

#### Validity

The values of average variance extracted (AVE) were used to determine convergent validity (Table 2 in Annexure). All the AVE scores are higher than the threshold value (0.5), and thus, convergent validity is ensured. Fornell-Larcker criterion and Heterotrait-Monotrait (HTMT) ratio were used to measure discriminant validity. Assessment of discriminant validity is a must in any research that involves latent variables for the prevention of multicollinearity issues (49–51). Fornell-Larcker criterion is the most widely used method for this purpose (49, 50). It compares the square root of the value of each average variance extracted (AVE) in the diagonal with the coefficient of correlation of latent variable (off-diagonal) for each variable in the related columns and rows (50, 51). A variable must explain the variance of its indicators better than the variance of other latent variables. Thus, the square root AVE of each construct must have a greater score than the correlations coefficient of other latent variables. In our case, the square root of each AVE of a construct is greater than the correlation coefficients of other constructs as shown in Table 4 in Annexure. So, discriminant validity is established as per the Fornell-Larcker criterion. Discriminant validity is also measured by Heterotrait-Monotrait ratio. To meet this criterion, values should be 0.9 or less. For this study, all the values are <0.9 (shown in Table 5 in Annexure), and hence, the criterion is met.

### Structural model

Latent variable correlation explains indicator reliability (50). Beta values indicating a correlational relationship among variables are significant (Table 1). Physical and chemical hazards are moderately correlated, while other variables show relatively strong relationships. As no coefficient of correlation is >0.8, the possibility of auto-correlation is ruled out.

**Table 1:** Latent variable correlation.

	**Physical**	**Awareness**	**Biological**	**Chemical**	**Ergonomic**	**Job Stress**
Physical	1					
Awareness	0.387	1				
Biological	0.352	0.476	1			
Chemical	0.578	0.419	0.487	1		
Ergonomic	0.251	0.525	0.289	0.276	1	
Job Stress	0.393	0.649	0.450	0.335	0.482	1

The value of R square explains that 42.5% variation in criterion variable is explained by exogenous variables included in Table 2.

**Table 2:** R square.

	**R square**	**R square adjusted**
Awareness	0.425	0.414
Job Stress_	0.421	0.418

The review of path coefficient (Table 3) shows that all the hypotheses have been substantiated except chemical hazards and job stress. It is evident that the majority of employees are aware of health hazards, and this awareness profoundly causes job-related stress. The coefficient indicates that 65% of occupational stress is explained by health hazards, and the *T*-value is 16.33, which is greater than the threshold point (1.96). The relationship is significant at *P* = 0.000. An ergonomic hazard is the highest stress in the model; 25% of job-related stress is caused by the ergonomic hazard. *T*-value (5.657) and *P*-value (0.000) substantiate the relationship. Biological hazards are the second-highest stressors after ergonomic hazards. *T*-value (4.80) and *P*-value (0.000) show that this relation is significant; however, the beta value (0.173) shows that the intensity of the relationship is not that strong. The hypothesis regarding physical hazards has barely been accepted. *P*-value (0.025) and *T*-statistics (2.245) substantiate the relationship, while the beta value (0.088) shows that the relationship is very weak. The hypothesis regarding chemical hazards has been rejected through all the indicators in the table.

**Table 3:** Path coefficient.

	**Original sample (O)**	**Sample mean (M)**	**STDEV**	**T statistics**	***P* Values**	**Decision**	**Nature of relationship**
Awareness -> Job Stress_	0.649	0.655	0.040	16.332	0.000	Supported	Positive
Biological -> Job Stress	0.173	0.178	0.036	4.807	0.000	Supported	Positive
Chemical -> job Stress	0.068	0.072	0.046	1.464	0.144	Not supported	No relationship
Ergonomic -> Job Stress	0.250	0.251	0.044	5.657	0.000	Supported	Positive
Physical -> Job Stress	0.088	0.093	0.039	2.245	0.025	Supported	Positive

As far as mediation is concerned, awareness mediates the relationship between health hazards (biological, ergonomic, and physical) and occupational stress, while mediation between chemical hazards and occupational hazards is not significant (Table 4). Since the direct relationship of biological, ergonomic, and physical hazards toward occupational stress was significant, however, due to the introduction of mediating variables, the degree of intensity of the relationship has increased. So, the author confidently affirms the existence of partial mediation. The beta value for ergonomic hazards has increased from 0.250 to 0.385; for biological hazards, the beta value has increased from 0.173 to 0.266; and for physical hazards, it has increased from 0.088 to 0.104 after mediation by awareness. In the case of chemical hazards, both the relationships (direct and mediated) remained insignificant.

**Table 4:** Specific indirect effect.

	**Original sample (O)**	**Sample mean (M)**	**STDEV**	**T statistics**	***P* Values**	**Decision**
Ergonomic -> Awareness -> Job Stress_	0.385	0.383	0.058	6.616	0.000	Supported
Biological -> Awareness -> Job Stress_	0.266	0.271	0.051	5.187	0.000	Supported
Physical -> Awareness -> Job Stress_	0.112	0.124	0.062	2.528	0.020	Supported
Chemical -> Awareness -> Job Stress_	0.104	0.110	0.070	1.495	0.135	Supported

## Discussion

With the support of literature and some hands-on experience, four occupational health hazards were selected for investigation regarding the given context. Literature exhibits quite deep stress among the workers in the dental health industry (52–55) that makes employees demonstrate unproductive or even counterproductive behaviors (56–58). In the same vein, literature regrettably affirms that large numbers of employees are unaware of occupational health hazards and their fatal consequences. Consistent with the Stress Concept Theory, Domino Theory, and ABC Theory, the model designed for the study at hand consisted of health hazards (ergonomic, physical, biological, and chemical), awareness regarding the prevalence of health hazards, and stress borne by the employees of dental hospitals. The services provided by dental hospitals are sensitive and important. Due to their relevance to human health, the quality of service cannot be compromised. The results of the study show that musculoskeletal (ergonomics) causes maximum stress among dental employees working in the selected hospitals. The chemical hazards have a minimum relationship with variables in the study despite previous studies indicating a significant relationship.

Exposure to chemicals such as formaldehyde, ethylene oxide, and antineoplastic drugs has caused many types of oncological diseases such as nasopharyngeal cancers and hematological cancer (29). Exposure to latex and other chemicals in disinfecting and cleaning is linked with work-related asthma (19, 59). Dental professionals are usually vulnerable to a variety of chemicals during their duty hours and may suffer permanent or temporary injury (60). Employees of dental hospitals mostly experience work-related eczema due to chemical irritation and allergies (61). Exposures to the chemical can initiate from dental materials, where reactive chemicals are released during preparation, polishing, and removal or restorations (62). Other sources of exposure are medical gloves containing rubber, chemicals allergenic latex protein, and different biocides/chemical disinfectants for infection control purposes (63).

The results of the study indicate that the chemical hazard attribute has a 1.04% (beta value) influence on stress at work with mediating role of employee awareness. The *T*-values <0.9 show that this variable has a minimum relationship with other variables in the study, despite some positive relationships between this hazard and stress found in the literature. The reason for this could be that the incidence of allergic reactions is less as allergic-free dental materials are now widely available and used in hospitals. Also, the subject under study dentists is not directly involved in handling chemical materials at the workplace. Many new advancements and research in dentistry have resulted in the formation of dental material with new chemical compositions. This could be the reason for fewer occurrences of chemical hazards in the population under study now.

According to Scully, due to the design of work and the equipment they use, dental professionals are at high risk of sharps injuries caused during any exposure prone procedure (EPP), where the employee's gloved hands can be in contact with the equipment in use, needle tips, or sharp tissues, e.g., spicules of bone or teeth (16). Results of the study showed that biological hazards have a beta value of 26.6%, and so, their effect on stress at work is more than chemical hazards. The reason for the strong relationship between biological hazards and stress is that dentists are more prone to getting infected by instruments as well as patients they treat. Dental professionals are directly involved in handling needles and sharps. The *T*-value of biological hazard is 5.18 (more than 0.9), which shows a strong relation between biological hazards and job stress.

According to the literature review, one comparative study by Rambabu on dentists showed that musculoskeletal diseases were found to be in high frequency among dentists than among other healthcare workers, and 60% of dental professionals reported complaints of more than one site (20). The results of the study show that the ergonomics attribute has a 38.5% (beta value) influence on stress at work with mediating role of employee awareness. Ergonomics or musculoskeletal injuries cause the highest level of stress among dental professionals. The *T*-value of ergonomics is 6.18, which shows a strong relationship between stress and musculoskeletal injuries. This shows that the working posture of dentists makes them prone to these injuries as they have to work in the same posture for long hours. Stress itself is the major cause of the development of musculoskeletal issues. Dentists are more prone to these injuries due to the nature of the job as well as stress. Previous studies in the literature found a strong association of stress with musculoskeletal hazards.

As most of the employees of dental hospitals possess medical knowledge and know the risk factors that exist in their work environment, the knowhow existence and prevalence of health hazards and their potential consequences create stress for the employees at work (64).

It is established that the theories used in the study provided sound bases, and the findings of the study enhanced these theories. The selected workplace hazards including ergonomic, physical, and biological hazards cause stress to the respondents, which depicts the application of Stress Concept Theory to the context as well as the respondents. The results of the study confirm the relevance of ABC Theory to the population under study, as the respondents were aware of the conditions where the existing health hazards had a different attitude and demonstrated a stressful behavior. There is a series of causes that eventually harm workers' health (Domino causation and control) like the existence of health hazards that cause stress and other mental disorders followed by compromised wellbeing. The workers actually experience stress as a result of the presence of health hazards.

### Contributions and recommendations

The study identifies the health hazards that exist in the work environment of dental hospitals. The composition of the dental healthcare working environment is not identical to other healthcare organizations. Work setting, posture to work, materials used, and nature of patients and their ailment are different from that of other hospitals, so the intensity of risk of health hazards is also different.

Ergonomic, biological, and physical hazards are more prevalent in the work environment that could harm the health workers. Apart from casting harmful effects on the health of workers, these hazards create stress in them. As most of the employees are educated and aware of the possible prevalence and risk of health hazards, consequently they suffer from stress. Ergonomic and biological hazards had severe prevalence, physical hazards had a moderate prevalence, and chemical hazards had minimum prevalence in these dental hospitals.

The study has significance in terms of its both theoretical and managerial implications. The study showed the least existence of chemical hazards, while literature portrays the otherwise. Due to certain structural interventions, chemical hazards have been reduced to a considerable level. The introduction of employee awareness as a mediating variable presents interesting findings. Employee awareness of workplace hazards makes them careful of keeping themselves safe from these hazards. At the same time, awareness creates job stress, and the stress itself negatively impacts employee wellbeing. On the contrary, unawareness makes employees carefree of workplace hazards falling prey to them. So, in the light of the results of the study, it is suggested that workers should be given enough awareness of the risk and dangers inherent in their work at the workplace, and through education, some of these accidents could be reduced if not eradicated. Jobs can also be designed in such a way as to remove all inherent potential dangers to make the work secure for employees. Management should work on both addressing workplace issues and creating awareness among employees regarding these hazards.

### Recommendations in brief

1. Ergonomic and biological hazards are intensely prevailing in the workplace and need some corrective measures.
2. Causes of the existence of ergonomic and biological hazards need to be explored to take corrective measures.
3. As stress is found among the respondents, it is necessary to adopt stress management strategies.
4. There is a need to make the hospital waste management system more effective. Improper disposal of wastes generates health hazards in the work setting.
5. Workers to ensure the complete observance of standard operating procedures and follow safety measures to avoid many health hazards.
6. Periodic training and workshops on workplace safety measures are to be conducted to enable workers to keep themselves safe from workplace hazards.

## Conclusion

The research findings reveal that health workers are exposed to occupational hazards that encompass biological needle stick injuries (viruses, bacteria, and parasites), chemical hazards (drugs and diagnostics), and ergonomic hazards due to poor body postures and irrational work programmed hours. Healthcare workers who encounter patients affected by HIV, TB, and Hepatitis B and C are exposed to these blood-borne infections. The results derived from the study indicate the higher prevalence of back pain among healthcare workers, in contrast to other occupational hazards. Consequently, the study emphasizes the need for organizations to address the issues associated with injuries occurring at the workplace by taking effective preventive measures. Substantial morbidity and mortality among these workers inevitably lead to the loss of skilled personnel, which adversely impacts healthcare services.

The research also brings out the analogy that victims of occupational hazards are more likely to encounter stress while at work. Resultantly, job-related stress is rapidly emerging as the major cause of work-related issues such as depression, anxiety, cardiovascular diseases, and stress-related disorders. The health sector at large and health professionals, in particular, are subject to these issues.

In short, workers irrespective of their field of work, when exposed to these vulnerabilities, inevitably fall prey to varying stress disorders. Hence, the research emphasizes the importance to address this stress-related issue as it not only adversely affects the smooth functioning of the organization but impede both patient care and service. The study shows that employees who are conscious of their surroundings are less prone to hazards and that leads to the fact that the key to preventing hazards is to know your surroundings, formulate policies and standard operating procedures, and periodic awareness training for hazard management. The outcomes derived from this study will supplement future research in this area. The study encompasses the source of hazards, the means to minimize and prevent the occurrence, and the realization of its importance among the health workers.

## Data availability statement

The raw data supporting the conclusions of this article will be made available by the author, without undue reservation.

## Ethics statement

This study was conducted as per the Ethical guidelines given in Helsinki Declaration. Written informed consent was obtained from all participants for their participation in this study.

## Author contributions

The author confirms being the sole contributor of this work and has approved it for publication.

## Conflict of interest

The author declares that the research was conducted in the absence of any commercial or financial relationships that could be construed as a potential conflict of interest.

## Publisher's note

All claims expressed in this article are solely those of the authors and do not necessarily represent those of their affiliated organizations, or those of the publisher, the editors and the reviewers. Any product that may be evaluated in this article, or claim that may be made by its manufacturer, is not guaranteed or endorsed by the publisher.
