# Supplementary material for: Awareness of occupational health hazards and occupational stress among dental care professionals: Evidence from the GCC region
Source: Front Public Health. 2022 Sep 8;10:922748. doi: 10.3389/fpubh.2022.922748 (PMC9493258; doi:10.3389/fpubh.2022.922748)
Supplement: Supplementary file 1 [file Table_1.docx]

**Annexure**

**Table 1.** Variables and items

| **VARIABLE** | **CODE** | **ITEMS** |
| --- | --- | --- |
| OCCUPATIONAL HAZARDS  EMPLOYEE AWARENESS  JOB STRESS | Chemical Hazards  CE1  CE2  CE3  CA1  CA2  CA3  Biological hazards  BH1  BH2  BH3  BH4  BH5  BH6  BH7  BH8  Ergonomic Hazards  MB1  MB2  MB3  MB4  MB5  MB6  Physical Hazards  PH1  PH2  WP1  WP2  WP3  WP4  WP5  WP6  WP7  SA1  SA2  SA3  SA4  SA5  JS1  JS2  JS3  JS4  JS5  JS6  JS7  JS8  JS9  JS10 | I wear eye shields/face masks for protection whenever there is a possibility of a splash  I attend to spills immediately as per spills policy  Colour-coded waste containers are readily accessible  I have suffered from a latex allergy  I report any allergies suffered at the workplace to my seniors.  Personal protective equipment is readily available  I dispose of sharp objects in sharps containers  I never recap used needles  I treat all materials that have been in contact with the patient as bio hazardous  I do not consume food or water in working areas  I follow appropriate waste disposal guidelines  I handle sharps with care as per ‘sharps protocol’  Colour-coded waste containers are readily accessible  Immunization for potential exposures such as Hepatitis B is mandatory  There are no physical obstacles in moving about in work areas  Work areas are not too crowded and cluttered  Housekeeping of work areas is satisfactory  I never suffer from back pains.  Repetitive tasks are kept at a minimum pace.  Sufficient rest breaks are provided to relieve stress from repetitive motion tasks.  There are safe wiring connections for machines, tools, and equipment at my workplace  All employees are required to report any hazard to life or property observed in connection with electrical equipment or lines.  Everyone receives compulsory health and safety training  Management is extremely particular about the certification  Systems are in place to identify and deal with hazards  Workplace health and safety are considered extremely important  There is an active health and safety committee  An incident report is filled with due diligence  Health and safety procedures are clearly communicated  I am clear about health and safety regulations at work  I have been certified by proper authorities  I know I can refuse to work in an unsafe environment  If I notice an incident at the workplace I am obligated to report it  I help my teammates understand the importance of health and safety  I feel that I am stressed at work.  I suffer from frequent headaches at the workplace  I suffer from anxiety attacks and depression  I have had this particular job stress< Than A Month,1-3 Months  4-6 Months, 6 months - 1 year, More than 1 year.  I would rate the level of job Stress as Mild, Moderate, Severe, or Extreme.  I get upset about workplace hygiene conditions  I am concerned about work environment safety.  Accidents often happen at my workplace.  The safety and health conditions where I work are good  Where I work, employees and management work together to ensure the safest possible working conditions |

**Table 2.** Reliability.

| **Construct** | **Cronbach's Alpha** | **rho_A** | **Composite Reliability** | **Average Variance Extracted (AVE)** |
| --- | --- | --- | --- | --- |
| Physical | 0.630 | 0.322 | 0.699 | 0.501 |
| Awareness | 0.865 | 0.871 | 0.894 | 0.516 |
| Biological | 0.721 | 0.681 | 0.802 | 0.507 |
| Chemical | 0.686 | 0.454 | 0.724 | 0.501 |
| Ergonomic | 0.726 | 0.703 | 0.794 | 0.511 |
| Job Stress_ | 0.756 | 0.731 | 0.752 | 0.531 |

Table 3. Variance Inflation Factor

|  |  | **VIF** |
| --- | --- | --- |
| Physical |  | 1.533 |
| Awareness |  | 1.284 |
| Biological |  | 1.365 |
| Chemical |  | 1.758 |
| Ergonomic |  | 1.132 |
| Job Stress_ |  | 1.628 |

**Table 4.** Discriminant Validity (Fornell-Larckers Criterion)

|  | **Physical** | **Awareness** | **Biological** | **Chemical** | **Ergonomic** | **Job Stress_** |
| --- | --- | --- | --- | --- | --- | --- |
| Physical | **0.647** |  |  |  |  |  |
| Awareness | 0.387 | **0.718** |  |  |  |  |
| Biological | 0.352 | 0.476 | **0.712** |  |  |  |
| Chemical | 0.578 | 0.419 | 0.487 | **0.686** |  |  |
| Ergonomic | 0.251 | 0.525 | 0.289 | 0.276 | **0.702** |  |
| Job Stress_ | 0.393 | 0.649 | 0.450 | 0.335 | 0.482 | **0.729** |

**Table 5.** Discriminant Validity (Heterotrait-Monotrait Ratio)

|  | **Physical** | **Awareness** | **Biological** | **Chemical** | **Ergonomic** | **Job Stress** |
| --- | --- | --- | --- | --- | --- | --- |
| Physical |  |  |  |  |  |  |
| Awareness | 0.801 |  |  |  |  |  |
| Biological | 0.897 | 0.619 |  |  |  |  |
| Chemical | 0.891 | 0.666 | 0.883 |  |  |  |
| Ergonomic | 0.550 | 0.654 | 0.392 | 0.456 |  |  |
| Job Stress | 0.887 | 0.885 | 0.746 | 0.683 | 0.700 |  |
